# Hints for a Medical Topography of Great Britain

**Published:** 1809-01

**Authors:** 


Medical and Physical Journal.

VOL. XXI.] JANUARY, 1809. [NO. 119.

*Printed for R. PHILLIPS, by W. Thorne, Red Lion Court, Fleet Street, London.*

### Hints for a Medical Topography of Great Britain.

### *In a Letter addressed to Dr. ADAMS.*

SIR,

THE extent and diversity of the operations of Nature, the fancied or the positive obscurity in her laws; the apparent, though probably only apparent, mutability in her facts, when placed against the short duration of human life, oppose a formidable barrier to the progress of medical science, and afford a brief but pithy commentary on the first aphorism of the father of the medical art.* At various periods, and for centuries together, these natural difficulties were increased by the improper, absurd, or ill-directed efforts of human intellect. *Galen*, and a legion of his followers, saw the face of Nature as through a magic glass, distorted into shapes that best suited to support a false, but imposing theory. *Paracelsus*, though he dispelled the illusions of the Galenic hypothesis, did but substitute the wild conjectures and bold assertions of an enthusiastic, perhaps insane chemist: and the mind was liberated from the persuasive eloquence and subtile reasoning of the Roman physician, only to be led into captivity by the incomprehensible jargon and dark visions of a barbarian of Switzerland. We have still to lament, Sir, that for fifteen centuries hardly a discovery was made, or a fact recorded, to which implicit assent could be allowed. During all this period the structure of the animal frame, and the laws of life were a blank;—the natural history of the *materia medica* was involved in fable the most absurd;—and the operations of medicines were governed by cold and moist, hot and dry, modified by an elaborate scale of degrees; or

* *Vita brevis, ars longa, occasio præceps, experientia fallax, judicium difficile.*

guided by an ideal intelligence under the name of *Archæus.* If the records of these facts were lost, and it were now asserted that the most enlightened physicians had been directed by this gallymaufry, the narrator would be considered rather as the libeller of the human understanding, than as the plain and faithful historian of actual transactions.

The causes that effected the destruction of systems that thus tyranized over true philosophy, were various; but we owe to our countryman, *Lord Bacon,* the direct means of removing such shackles from the understanding, and of investigating Nature on the principles of a genuine science. The *Novum organon* explained the advantages and the modes of an experimental philosophy, unknown both in name and practice to former ages. From that period facts have been gradually accumulating. The laws that govern matter in all its modifications, animate and inanimate, have step by step been evolved, until a knowledge of the visible creation has approached to a demonstrative certainty. If within the last century, natural history in all its branches, chemistry, botany, materia medica, practice of physic and surgery, the laws, composition and impregnations of the atmosphere, &c. have assumed a form and precision almost to satisfy the most fastidious; we must not admit, however, that no more facts remain to be brought into the depositaries of learning, or that science has reached its *ne plus ultrà.* Daily experience points out that much is yet left to be done; and the discovery of new facts, and the developement of new principles, constantly instruct us, that systems deemed most complete, and arrangements thought sufficiently comprehensive and correct, are yet far from absolute perfection.

If medicine is the science of observation;—if its principles are founded on experience, and never to be inferred *a priori,* by any depth of research; the only method by which it can be brought to perfection, is that so distinctly indicated by *Bacon.* A studious and diligent inquiry into every circumstance, directly or collaterally connected with animal life;—into the properties, whether medicinal or deleterious, of animals, plants, and minerals;—into local peculiarities, either accidental, fugacious, or fixed;—and indeed into almost every condition of external Nature, becomes then the serious duty of the medical practitioner.

Fully impressed with the cogency of this conclusion, and of the advantages to be derived to the science of medicine by steadily pursuing the path of *experience* and *observation,*

*tion*, I have ventured to avail myself of the means so liberally afforded by the Editors of the Medical and Physical Journal, to solicit the attention of the Faculty of Great Britain, to inquiries that I am willing to hope may be of service to science generally; and finally, perhaps, produce materials for a *Medical Topography* of the BRITISH ISLANDS.

An intelligent and philanthropic writer in your Journal for last September, has requested a communication of popular opinions and received notions relative to the origin, cause, prevention, and cure of diseases; and also of the composition of efficacious nostrums, differing essentially from the *formulæ* of regular pharmacy. Every person can understand that such communications may benefit society, either by destroying the groundless fame of some vaunted remedy; or prove the propriety of adding others to the general stock of knowledge, by rationally ascertaining their efficacy. But the principle of *common sense*, directed by medical science and a general acquaintance with natural history, may push on the inquiry to investigations of great importance.

The volume of Nature lies open to the medical philosopher: in all regions he is excited to peruse her pages, to interpret her language, and explain her laws. In the expeditions, whether of discovery, war, or commerce, made to remote parts, and into every climate of this earth, we see with a pardonable satisfaction, that the most interesting facts in natural history and science, have been collected by medical persons; or by those educated in that profession. It seems to me, Sir, that a spirit of inquiry into the natural phenomena going on in this globe, is congenial with the study of the medical art; and that this disposition may be encouraged and directed in a mode that may lead to important discoveries, and to the acquirement of a most precise knowledge of every country and people. But in this laudable task let us begin at HOME.

There are, I apprehend, unrecorded circumstances of much interest in natural history and medicine, daily occurring in the British Islands; as well as local peculiarities connected with these circumstances, either unnoted or not understood. It will be admitted, that the origin and course of disease often bears a close relation to these local peculiarities. Influences of the atmosphere, gratuitously assumed without understanding their properties, are said to affect, very extensively, the health of mankind. Exhalations from the earth or waters; mineral effluvia; vegetable aroma; direction and degree of winds; accumulations of electricity, whether silently operating or bursting in storms; modes of life in towns and in the country; effects produced on health in societies pressed together in manufactories, or separated in agricultural employments; the indigenous origin or importation of contagion; the causes of epidemics; the properties, operations, and final effects of many substances taken in food or medicine, or exhibited as poisons, are yet imperfectly understood, and require that legitimate investigation, once employed by *Hippocrates* and *Sydenham.*

But in estimating the value, whether commercial, literary, or scientific, of numerous and difficult subjects, it is essential to pursue a regular method; and I presume, Sir, on the indulgence hitherto granted me, to attempt the detail of a plan, which if I cannot expect to be generally admitted, I may hope will be allowed by all to be given with the desire of making better known the state of medicine and natural history in our own country. To obtain this, as I conceive, desirable object, nothing more seems required than to excite the Faculty dispersed over the kingdom, to record and make known local facts in medicine, and the sciences connected with it; to give the natural history of the places where they are stationed, until by degrees a stock of materials is collected for a full and complete topographical and scientific history of the *Medical Art.*

In the particularization of the points which have occurred to me as being useful in the prosecution of this inquiry, but which are to be considered, however, as merely suggestions submitted to the Faculty, I have adopted the subsequent arrangement, as being sufficiently comprehensive to include generally the facts applying to this subject.

1st. Local peculiarities, not immediately arising from inhabitants or productions.

2d. Natural History, including Animals, Vegetables, and Minerals.

3d. State of Medical Practice, Diseases, Veterinary Art, and Empiricism.

In the small space occupied by England and Wales, (and at present I do not wish to extend the Inquiry beyond that portion of the British islands) if no variation of climate, properly so called, is to be found, some difference of temperature certainly there is between the coasts of Cornwall and the bleak hills of Cumberland; some diversity of elevation and depression of surface from the summits, summits of Snowdon to the low-lands of Cambridgeshire. These accidents of situation, perhaps, connected with habits of life, certainly influence health in no inconsiderable degree; for it cannot be denied, that trains of disease exist in some parts, never heard of in others. The *Flora* of different districts will also be found to vary very considerably. In some parts the only ascertained poisonous reptile multiplies in great abundance, while in others it is never seen. The eagle that inhabits the rocks of Borrodale, is never met with in the mid-land counties but when driven thither by some casualty. These are, however, but hints to indicate the varieties that Nature exhibits in her works within a circle of a few hundred miles; and cursorily to point out the diversity of facts that render a minute investigation desirable.

In prosecuting this inquiry, that general division of the subject which is intended to explain LOCAL PECULIARITIES, NOT ARISING FROM INHABITANTS OR PRODUCTIONS, claims a priority of discussion, as probably affording the principles that give colour and character to natural history, and influence, in a degree, diseases, and modes of medical practice.

*Local peculiarities,* under the circumstances indicated in the title of this general division of the subject, will comprise distance and direction of the district or town from the metropolis; elevation above the sea; uniformity or irregularity of surface; character of soil, clay, sand, or bog; relative quantity of pasture, arable, and wood-land; state of drainage, whether the water is rapidly run off, or suffered to stagnate and be gradually exhaled into the atmosphere: source and direction of rivers; state of lakes and morasses; temperature, and mean height of the barometer;* quantity of rain, and the extreme points of humidity in the atmosphere. The quality and degree of electric fluid, as detected from time to time by the electrometer, or manifested in storms: with the effects produced on the air by its violent actions. In those districts where mines are found, to ascertain their products, describing particularly those believed to affect the health, or afford substances for the materia medica; pointing out the appearances of the

 strata,

* Where there is great difference in elevation, as in the vale of Keswick and the summit of Skidda, an estimate of the relative states of the mercury, under different circumstances of the atmosphere, may not be matter of mere curiosity.

strata, and the effects produced on the surface of the earth by the contained mineral. The importance of respiration to animal and vegetable life, and the action of air upon every substance susceptible of change, render all inquiries into the actual degree of purity in the atmosphere, and into its usual or accidental impregnations, as connected with, or influencing the state of health and disease, a most urgent duty of the medical practitioner.* By ascertaining the real qualities of these impregnations, and pointing out their cause and origin, incalculable benefits may arise to society from the elucidation that the sources of contagions and epidemics will, possibly, thus receive. Another circumstance connected with this head of the inquiry, is the history and description of mineral springs; minutely pointing out the chemical contents, medical effects, and actual state of such as are but little known.

This detail, to which perhaps every person applying to the subject will suggest additions, is properly followed by the Natural History of the Animal, Vegetable, and Mineral Kingdoms.

Britain is neither remarkable for the production of the powerful and sanguinary among quadrupeds and birds; for the venomous among insects and reptiles; or for those which afford substances for the materia medica. The larger species of quadrupeds and birds scarcely possess any properties that connect them with the Pharmaceutical Art; and perhaps, with the exception of the *ursus meles,* there is not one among the genera of these creatures that affords any active material for medical use. That animal has an apparatus for the secretion of a substance so analagous to musk, that it should not, perhaps, be entirely overlooked.

So fortunate is England, that few, if any, individuals of the first great division of nature are here found, decidedly and naturally possessed of that degree of virulence, either in their bite or any other property, constituting poison. The viper (*coluber berus* of Linné) is the only species of the tribe of serpents that has with any degree of precision, been ascertained to have a specific venom. The *Anguis fragilis,* which is now and then met with in some districts, has been accused of possessing poisonous properties; but it has this character, perhaps, from the bold manner in which it attacks, coming almost erect, and apparently fearless, to encounter its adversary. It has been suggested that there is a variety of the viper, distinguished by a considerable increase in size, and known by the popular term

*adder.*

*adder*. The viper exists principally in the wooded highlands of Essex, Suffolk, and other mid-land counties, in dry, stony, and particularly chalky soils:* the adder, if such a variety there is, lives in marshy low-lands, and bogs. A man removing turfs or peat, dug for fuel in the fenny parts of Cambridgeshire, was wounded in the hand by a snake which lay coiled among them. In an instant he destroyed the reptile, but was himself almost as suddenly affected with excessive pain and tumour in the wounded hand. From the rapid increase of inflammation, from the extreme morbid derangement of the circulating system, and from the affection of the brain and nerves, manifested in delirium and *subsultus tendinum*, his life was put in great danger. He, however, recovered. This snake had the external appearance, but was double the size of the largest viper. Are we as well acquainted with the Natural History of the serpents of England and Wales, as with those of India? It is true, that none of that genus exist here with the deadly properties of the *cobra di capello*; but the viper has generally been considered as secreting a fluid with such deleterious powers, that the insertion of a small particle of it into wounds, was connected with great danger, and often followed by death. There has been a notion, however, that the whole of our opinions respecting the poisonous bite of the viper, are false; that it is perfectly inoxious, and that every thing which we have heard with respect to the utility of olive oil in the bite of this reptile, has been founded on a mistaken notion: and that the history of *Oliver* the viper catcher, was on his part an expert piece of legerdemain; and an instance of remarkable credulity on the part of those who witnessed the deception. If we are yet to learn, whether the bite of the viper is or is not poisonous, our knowledge of the Natural History of our own country is at a very low standard.

Among the insects of Britain some will be found to possess fluids highly stimulant, and, perhaps, now and then occasioning death; and though they are not virulent in the same degree with the *Furia infernalis*, the *Pulex penetrans*, the *scorpion*, and the *tarantula*; yet their natural history, and the instances of mischief arising from an application of their venom, are not unimportant. Of the genus *Vespa* we have three species, all of them possessing the

* In some of the Hebrides the breed of vipers is said to be extremely numerous.

the property of producing violent and painful inflammations, sometimes followed by considerable danger, when the injury has been inflicted on parts of great sensibility, and in peculiar idiosynscracies. The *Apis mellifera*, whose œconomy is so well understood, has, when attacking in multitudes, in a few instances, been destructive of life in the larger animals. A genus of flies, the natural history and properties of which have been lately investigated, in the Transactions of the Linnæan Society, with much ingenuity by Mr. Clark, deserves great attention. The *œstrus equi, bovis,* and *ovis,* produce such singular effects on horses, sheep, and oxen, that their natural history is not only a matter of curiosity, but an object of importance in rural œconomy as well as science. In many parts of the world, it is fully ascertained that an almost imperceptible insect of the genus *Acarus,* produces an extremely troublesome disease of the skin. In Germany, Italy, Madeira, some of the islands of the southern hemisphere, and even in Ireland, the existence of the *Acarus exulcerans* has been demonstrated. That it has not yet been seen in England, is, perhaps, more owing to want of dexterity in the search, than from mankind being here free from the scourge that this minute creature inflicts. In the *Systema Naturæ,* the genus *Acarus* consists of more than eighty species: upwards of twenty of these species live on the cutis of various animals, which they unceasingly torment, and sometimes destroy. Various scarabæi are devoured by them. *Donovan* gives a very correct figure of the *Acarus coleoptratorum* feeding on the *Scarabœus stercorarius.*

Few of your medical readers, Sir, will be found who have not witnessed the extraordinary symptoms that have suddenly arisen on eating the muscle. An efflorescence, resembling scarlatina, has, after such a repast, suddenly spread over the whole surface of the body, accompanied with a universal tumour of the extremities and trunk. In the instances which have occurred to my observation, the morbid appearances have soon vanished, after evacuating the poisonous material from the stomach. Perhaps the seas that surround our shores, afford more instances of deleterious properties in fish; and it should make a part of the survey of the maritime counties, to state the instances in which such effects have occurred; the symptoms, treatment, and termination of the cases; and a precise identification of the species of fish occasioning such appearances.

This first department of Nature in England, affords but one substance for our *Materia Medica.* The *Oniscus asellus* still retains a place in the London Pharmacopœia. The viper, and its preparations, which our forefathers dignified with such powers of restoring debilitated nature, has sunk into that disrepute which it probably deserves. It may be right, however, to enquire if it is still used medicinally in any form. A short time since we were amused with the relation of the extraordinary and almost magical effects of the *Cocinella septem punctata,* in tooth-ach. The discovery, I think, was made in Germany or Italy, and detailed here with some confidence. As no instance of its success has occurred to me, or any of my friends, I should not now have noticed it, but to observe, that in the autumn of 1807 such myriads of this beautiful little insect landed on the shores of the county of Kent, especially in the neighbourhood of Margate, that the ground was enamelled with them, and a foot could not be put down without crushing multitudes. As far as my information extends these vast numbers came across the Thames from the opposite shore of Essex.

The paucity of facts that connect the animal kingdom with the *materia medica* of this country, will be fully counter-balanced by the abundance of native vegetables possessing either medicinal or deleterious properties. The Pharmacopœia of the London College admits more than forty indigenous plants; and many others are found, having active qualities deserving investigation, though not yet so correctly determined as to establish their character. The examination of these may lead to the formation of a *Flora* of every district from actual observation. In making out descriptive catalogues of the plants of any given place, it does not seem, however, so much an object to comprehend every individual plant, as particularly to remark, and correctly describe those which are admitted to possess either medicinal or poisonous qualities; and to ascertain by experiments the real properties of others, to which popular report annexes extraordinary powers. In the prosecution of this inquiry it will, perhaps, be deemed proper to give most attention to those plants, whose properties are so far ascertained as to gain them admission into the College Pharmacopœia. This certainly will be right, inasmuch as it goes to the developement of any quality in them not yet known, or to a more precise ascertainment of their admitted medicinal powers. But as the avowed object of this inquiry is to bring forward every latent or obscure fact relative to the preservation of health, and the cure of of disease, it will follow, that in making out the local *Flora*, a careful attention should be given to the provincial and vulgar names of plants, in a manner that may readily refer them to the scientific nomenclature; that an anxious solicitude should be exerted to explain their popular virtues, and the common or country methods of exhibiting them as medicines. In this class of nature euporistic medicine will be most generally found. Among the common people there are many remedies usually taken from the vegetable kingdom, easily procurable and readily prepared; and of considerable efficacy. These sometimes contain substances of great activity, and when misapplied, either in dose or intention, of extreme danger. But whatever their properties or powers may be, it is surely right to have them clearly understood.

In the arrangement of the *Flora* of medicinal plants, it will be proper to remark those situations where they are produced in great abundance: and if in such places their qualities seem improved, or their powers remarkably elaborated, to ascertain with the utmost precision the peculiarites of the soil; the aspect, whether exposed or sheltered; and if growing in woods, to point out the species of trees that shade them. Where medicinal plants are cultivated, it will be an object of some interest to detail the modes of culture, and to what extent the produce goes, as well as the manner of disposing of them when prepared for sale. On this subject, the cultivation of the poppy, with a view to the manufactoring of opium, cannot pass unnoticed. The quantity and quality of the opium thus produced, the methods of manufactoring it, and the actual or probable advantages that are or may be thus derived to the nation, is a subject of obvious inquiry. Some species of the genus *salix* have been confidently recommended as a powerful remedy for intermittents; and the bark of the *salix latifolia* has been particularly pointed out as possessing powers little short of the *cinchonv.* With this character it cannot but be desirable to ascertain the best mode of preparing it for use, the form and quantity of its dose, and its final effect: whether there are any districts in which it continues to support its reputation as a febrifuge; or whether it has been applied, and found efficacious as a tonic in dyspeptic states of the stomach, unaccompanied by fever.

In the catalogue of the medicinal plants of Britain, there are enumerated some of extraordinary efficacy. The genus *Solanum* affords two species, the *Atropa belladona* and the the *Solanum dulcamara*, have properties yet, perhaps, not fully ascertained. The *Conium maculatum*, the *Hyociamus, Colchicum autumnale, Digitalis purpurea, Daphne mezereum*, and *Helleborus fœtidus*, are also powerful narcotics or stimulants of a peculiar kind, whose *modus operandi* still remains obscure. The total failure of the narcotic vegetables in this country, when compared, at least, with the effects asserted to be produced by them on the continent, is a fact too remarkable to be silently passed over. That, they act with great power upon the animal system, even to a fatal degree, is well known; but their property of curing the most formidable diseases, as seen in the relations of *Storck* and others, is not in the least commensurate with their evident effects, or with the promises of the German physicians. An inquiry into the cause of this contrariety is certainly a duty. Does it arise from the lowered quality of the plants, the effect of climate? This should not be the fact, for here they are powerful enough to destroy life. Is it from want of skill in their preparation or exhibition? This we are not disposed to admit. Are the extraordinary effects produced upon disease, especially *carcinoma*, by the *conium*, as related by *Storck*, to be considered as extravagant, exaggerated, or false? Though we are not able to produce similar effects with this material, let us have the candour to forbear such charges, till every mode of trial is exhausted. I am aware, that a lapse of fifty years may be considered as having determined this point. But scepticism and credulity are equally to be avoided. That these narcotic plants possess powers potent to extinguish the vital principle, no one denies; can it then be doubted that they also possess the property of operating on the actions of this principle in various modes and degrees, proportioned to the quantity exhibited; or to the sensibility to their impression in various idiosyncracies? As this tribe of plants evidently have great influence upon the sensorium, and through it upon the actions of the living principle, generally modified by shades of difference in each of them, it is essential to call into the investigation the opportunities, the skill, and the sagacity of the faculty at large. Almost every practitioner has seen, that the soothing quiet which is sometimes refused to opium, has followed the employment of cicuta; where this has failed, hyosciamus has produced the desired effect. In this particular the observing practitioner must have detected much variety as well as the creation of new remedies by combinations o[illegible] hese narcotics. But if we have have not yet arrived at the nicety of discrimination that enables us to say upon any given occasion, which of these narcotics, or of these combinations, is the medicine required, we have much to learn.

Those who have considered the visible creation with the eye of science, or the feeling of philosophy, either in the aggregate or in detail, cannot but have observed with what beneficent views, and with what perfect skill, the parts are adjusted; and with provident care adapted to the wants of the whole. The powerful remedies we have just noticed, are not to be sought for in remote regions, under the hazard of precarious supplies; but are presented to us in every field or hedge-row; scarcely a bank or a wood, but affords them. Are we not called then by the voice of Nature, to persevere in the examination of the properties of these common simples, until their medicinal powers are more certainly developed, and their action upon the animal system better understood?

Besides the plants before mentioned, the London Pharmacopœia admits into its *materia medica* many other natives of Britain; several of them are of known efficacy, some are doubtful, others approach to inertness. There are also no inconsiderable number not admitted into the class of established remedies, which possess powers of sufficient activity to attract notice. The genus *ranunculus* affords some species extremely acrid and stimulating. The *R. sceleratus* and *acris,* are even corrosive. The former of these is used by beggars to produce ulceration; and the latter, when taken into the mouth, blisters the tongue. The *R. arvensis* poisons sheep, and three ounces of the expressed juice killed a dog in four minutes. The acrid principle of the *R. flammula* rises in distillation, and affords an emetic that operates the instant it is swallowed. That it operates instantaneously, was asserted by the late Dr. Withering from his own knowledge of it, and that without exciting those painful contractions at the upper orifice of the stomach, so often happening when the sulphate of zinc is used. In cases of poison, such a remedy as this must be extremely valuable. In general, cattle refuse the species of this genus in their green state; when, however, they chance to be eaten in a moderate quantity, they occasion bloody urine, and other effects of an acrid stimulant. Some species of *Anemone* have the same effect; especially the *nemorosa,* which also occasions dysentery in sheep. The *Bryonia dioica* certainly deserves more attention than has been given to it. It is an active cathartic, either in substance substance or infusion; and probably, a tincture made with wine or proof spirit, would be a useful officinal composition. The root also affords a rubefacient, and a cataplasm which has the character of being a powerful discutient. A decoction of the fresh root is an active purge for cattle. An infallible cure for the *Crusta lactéa* is said to be found in the *Viola tricolor,* which grows abundantly in some of our corn fields. The *Daphne laureolæ* is an active, perhaps severe carthartic, and an efficacious vermifuge; singularly happy effects are said to have been experienced from it in acute rheumatism. *Cynoglossum, Datura stramonium, Solanum nigrum, Marrubium,* also deserve investigation. With the latter of these Linneus cured a mercurial salivation of long standing, and which had resisted every other means. The leaves of the *Hedera helix,* vomit and purge; and Haller asserts, that they have been given in Germany as a specific in the atrophy of children. Here I might add a multitude of instances to illustrate this subject; but what has been said may suffice for a specimen of the opinions that have, or do still prevail, with respect to the medicinal properties of many indigenous British plants. And though implicit faith should not be given to popular opinions upon medicine and science, yet those opinions should not be despised, but rather excite to investigation that may fully decide upon the alleged medicinal virtues of our native vegetables.

It does not appear to accord with the benevolence of Providence, that such numbers of poisonous vegetables should have existence. But this objection vanishes, if we admit with the greatest naturalist that ever lived, that every poison is a concealed medicine. If this is to be credited, exertion should be redoubled to call forth the latent good from the obvious evil. Upon this principle, we are to consider that not only the narcotics we have enumerated, but that *Œnanthe crocata, Cicuta virosa, Phellandrium aquaticum, Solium temulentum,* and many others, have in them concealed medicinal properties quadrating with their deleterious principle.

How has it happened that no attempt has been made to discover some medicinal principle among the 500 species of *Agaric* that indigenously vegetate in our pastures and woods? We have often heard of their poisonous qualities; and a very distinctly marked case in your Journal, for November, adds another fact to the fatal catalogue: but no instance has occurred of their having been thought applicable to the cure of disease. Some cases of the deleterious species species being taken into the stomach, have fallen under my observation. In those, the effect on the *sensorium commune* was strongly marked. Wild, but not furious delirium; slow and feeble pulse; tremor; *subsultus tendinum;* and a singularly expressive character of intoxication, were the symptoms that indicated the *modus operandi* of the poisonous *Agaric*; and by those who recovered, an interesting account was given of the recollected intellectual derangement. It may not have occurred to all your readers, that the *Agaricus muscarius,* a species not uncommon in our pastures, is employed in the north of Europe for the same purpose that opium is taken in Turkey, and bange in Hindoostan. The Russians, the Kamtschadales, and the Koriacs, use it as an instrument of intoxication. They sometimes eat it dry, sometimes take it mixed with a fermnted liquor, made with the *Epilobium.* Soon after swallowing this potent substance, they are described as being seized with convulsions in all their limbs, then with furious delirium: a thousand phantoms, gay or gloomy, are presented to their imaginations; some dance, others are seized with unspeakable horrors. These barbarians personify this vegetable under the name of *Moucho-more;* and if its effects urge them to suicide, or any dreadful crime, they say they obey its commands. To fit themselves for assassinations, they take a full dose of the *Moucho-more.*

It is not easy to calculate to how many purposes the active imagination of man has applied the narcotic plants. Medicines, poisons, intoxication, and madness, lie concealed among their juices. It is the duty of the physician to discriminate their beneficial effects, to teach mankind to know them as poisons, and to avoid their habitual use, however soothing to the feelings, as the baneful source of weakness, paralysis, and fatuity.

The mineral kingdom gives some of the most powerful and active articles to the *materia medica.* From this division of Nature are taken antimony, arsenic, mercury, lead, copper, iron, tin, sulphur, &c. Perhaps every mineral substance in our *materia medica,* with the exception of mercury, may be procured in Britain, in quantities amply sufficient for the purposes of medical practice: we do not import antimony and sulphur because they are not to be found at home. Cornwall, Derbyshire, and Wales, abound with mineral substances; and in them metallurgy may be studied with every possible advantage that Nature affords. These districts present to the chemist, the medical philosopher, and the naturalist, the most ample means of gratification; gratification; and no doubt can remain, but the medical practitioners who reside in the mining counties, did they dedicate a part of their leisure to this inquiry, would bring into the general stock of medical knowledge many facts of great interest and importance.

The last part of this Inquiry is intended to incite to an investigation of the

State of Health and Disease, of Medical Practice, of the Veterinary Art, and of Empiricism.

It is a generally admitted fact, that the inhabitants of some places have more perfect health than those of others; that the term of life is much contracted in some, and in others much extended; that diseases of one type occur in this, and of another in that place. These effects have their corresponding causes; and it is a *desideratum* in the science of medicine to establish those causes on the basis of experiment and observation.

It is a primary consideration in this Inquiry, to ascertain beyond dispute, whether there are certain districts where the state of health is absolutely more perfect than in others. And this not in particular years or seasons, but generally, and in all constitutions, as it has been quaintly called, of the atmosphere. Whether, upon all occasions, endemial complaints, epidemic and contagious diseases, have been altogether absent or less frequent than in other districts. A general view of the history of health, the average number of deaths compared with the population, and instances of longevity, will go far to establish the fact of this reputed privilege.

It is at least a popular notion, that the greater the elevation the more perfect the state of health. The mountaineer is commonly characterized by a robust energy of frame, and length of days, denied to the inhabitants of vallies. But the scientific enquirer will refer to a number of circumstances to account for this privilege. Peculiar localities, noticed in the first part of this Inquiry, will not escape his observation. There may likewise be operations going on in the economy of vegetation, not unlikely to influence the salubrity of places. This happy state of exemption, or partial exemption from disease, will also depend much on modes of life; on diet, exercise, and temperance; on employments, whether sedentary manufactories, either solitary or gregarious; mining, chemical processes, or agriculture. It will be found that there is some difference in the duration of life in large towns, and in villages and farms.

After

After having clearly and fully ascertained the interesting *fact* of the inhabitants of some places being more free from *disease,* and affording instances of long-life, in a greater proportion than others; a sedulous inquiry should be instituted to ascertain with all possible precision, the direct and incidental causes giving rise to this superior healthiness. These causes may be referred to two classes. The first class will arise altogether out of local peculiarities; the second must be referred to the state of society, and habits of life.

Many of the first class of causes have already been pointed out. The elevation, qualities of the soil and water, and the changes in the district affected by the operations of human industry, should be examined with great attention; and the actual and usual state of the circumambient air should be perfectly elucidated.

It must have occurred to every person who has at all reflected on the progress of medical opinions, and on the endeavours to ascertain the causes of endemial and epidemical diseases, that recourse has generally been had to some peculiar state of the atmosphere. From the earliest records of physic to the present time, though the cause of these diseases has been sought for in some change or impregnation of the ærial fluid in which animated nature is constantly immersed, has it ever been satisfactorily ascertained what the quality of that impregnation, or the nature of that change has been? Pestis, epidemic catarrh, fevers of almost every type, remittents and intermittents, and even syphilis, have at times been referred to an atmospherical influence. But has it ever been ascertained with sufficient exactness, even in those diseases most likely to arise from some peculiar quality of the air, whether the state of the atmosphere was the same in all of them? or whether, at any time, it differed in the proportions of its usual combinations? was more rare or more dense? whether it had any new and uncommon impregnations? was of a very high or very low temperature? or whether it was actually the same as in the most healthy seasons? During the prevalence of epidemic diseases, or of those suspected to be contagious, the importance of ascertaining the real properties of the atmosphere will be readily admitted.

( To be concluded in our next. )

